# Pathophysiological Role and Therapeutic Potential of Vitamin C in Metabolic Syndrome and Type 2 Diabetes Mellitus

**DOI:** 10.3390/metabo15120773

**Published:** 2025-11-28

**Authors:** Christiano Argano, Valentina Orlando, Dalila Maggio, Chiara Pollicino, Alessandra Torres, Virginia Cangialosi, Stefania Biscaglia Manno, Salvatore Corrao

**Affiliations:** 1 Department of Internal Medicine, National Relevance and High Specialization Hospital Trust, ARNAS Civico, Di Cristina, Benfratelli, 90127 Palermo, Italy; 2 Department of Health Promotion Sciences, Maternal and Infant Care, Internal Medicine and Medical Specialties [PROMISE], Univeristy of Palermo, 90133 Palermo, Italy

**Keywords:** vitamin C, type 2 diabetes mellitus, metabolic syndrome, pathophysiology, oxidative stress, antioxidant, therapeutic potential

## Abstract

Recently, a growing interest has been focused to the role of vitamin C in chronic diseases. Type 2 Diabetes Mellitus and the Metabolic Syndrome are complex, chronic disorders intrinsically linked by a common underlying element, such as chronic low-grade inflammation and excessive oxidative stress. Vitamin C, or ascorbic acid, is an essential water-soluble micronutrient and a highly potent non-enzymatic antioxidant that is critical for scavenging reactive oxygen species and maintaining cellular redox balance. It represents a cofactor for many enzymes, being involved in many biological functions, such as normal immune system functioning, catecholamine metabolism, dietary iron absorption, and collagen biosynthesis. Individuals with type 2 diabetes mellitus and metabolic syndrome frequently exhibit lower circulating and dietary vitamin C levels compared to healthy controls, a deficiency that may be associated with disease-related inflammation and higher body weight. In this sense, it has been shown that vitamin C improves skeletal muscle insulin sensitivity in experimental settings and modulates critical functions like vascular endothelial health. However, this potential is challenged by the fact that chronic hyperglycemia can interfere with the active cellular uptake and transport of vitamin C, potentially leading to relative intracellular deficiency in diabetic patients regardless of intake. It is interesting to note that different studies have demonstrated an inverse relationship between vitamin C concentrations and the prevalence of metabolic syndrome and type 2 diabetes. Vitamin C supplementation in people with diabetes and metabolic syndrome has controversial effects. While several studies indicate a significant reduction in fasting blood glucose or HbA1c, others revealed no significant effect on insulin resistance. This review aims to explore the pathophysiological role and therapeutic potential of vitamin C in type 2 diabetes and metabolic syndrome.

## 1. Introduction

Diabetes mellitus (DM) affects nearly 600 million people worldwide, with type 2 diabetes (T2DM) accounting for around 95% of cases [1]. T2DM causes significant cardiovascular and kidney-related morbidity and mortality [2], which results in a relevant healthcare burden and hospitalization [3]. A holistic approach is crucial for complex patients with cardiac, metabolic and renal diseases, especially those with diabetes. Targeting blood pressure, lipid levels and insulin sensitivity can reduce the risk of cardiovascular disease in T2DM. Metabolic syndrome (MetS) refers to the clustering of at least three of the following five metabolic conditions: increased waist circumference; high blood pressure, fasting glucose levels and triglycerides; and low levels of high-density lipoprotein cholesterol [4]. MetS is a disorder that drives the twin global epidemics of type 2 diabetes and cardiovascular disease [5]. Those with diabetes are three to five times more likely to have MetS, which is now common in many countries. Worldwide, 7.9–43% of males and 7–56% of females have MetS. MetS is influenced by several factors, some of which can accelerate disease progression and lead to various complications and aggravate MetS-related morbidity [6]. MetS is recognized as a significant public health concern, along with obesity and diabetes [7].

A shift in diabetes management from a glucocentric approach to one more focused on reducing these complications was facilitated by the rapid emergence of novel effective medications, including sodium-glucose cotransporter-2 (SGLT-2) inhibitors and glucagon-like peptide-1 receptor agonists (GLP-1RAs) [8]. Evidence regarding the weight-lowering effects of GLP-1RAs and other medications such as tirzepatide is also attracting global attention consequently finding application in metabolic syndrome [9,10,11]. Additionally, a different medication, finerenone, has demonstrated kidney benefits in subjects with established chronic kidney disease both alone [12] and in combination with SGLT-2 inhibitors [13]. This provides physicians with another tool against type 2 diabetes. However, variability in access to medications and related costs also impacts international decision-making [14]. The use of vitamins has gained significant attention in recent years, particularly during and after the SARS-CoV-2 pandemic. The pathophysiological factors underlying impaired glucose metabolism and vascular complications of T2DM include excess reactive oxygen species and oxidative stress [15,16]. Several studies have also documented that a decrease in antioxidant levels and an increase in inflammatory and oxidative stress biomarkers may be involved in the pathophysiology of T2DM complications [17] and the onset of cardiovascular diseases, suggesting that antioxidant therapy may be effective in improving glycemic control and cardiovascular risk factors in people with T2DM [18,19,20,21,22,23,24].

**Figure 1:**
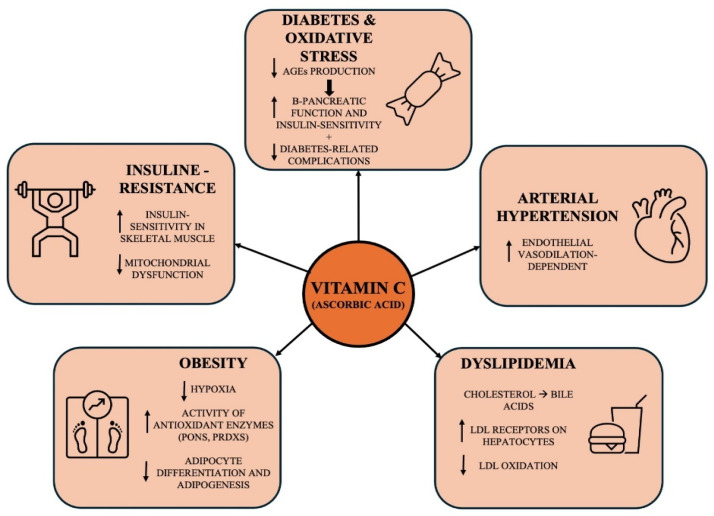
The Pleiotropic Role of Vitamin C in Diabetes and Metabolic Syndrome.

In this regard, there is growing interest in using vitamin C. Also known as ascorbic acid, vitamin C is a water-soluble vitamin that works as a strong antioxidant and is involved in various biosynthetic pathways within the immune system. It is an essential nutrient that the human body cannot produce itself [25]. Its antioxidant properties are derived from its ability to donate electrons, which helps protect molecules from oxidative damage. Vitamin C has been investigated for therapeutic use in various critical and non-intensive care conditions, with mixed results concerning laboratory and clinical outcomes [26,27,28,29,30,31,32]. Vitamin C has also been studied as a potential treatment for T2DM. While T2DM is not the only condition in which vitamin C supplementation may be beneficial, previous systematic reviews of randomized controlled trials (RCTs) have focused on its potential to improve glycemic management [33], blood pressure (BP) [34], and endothelial function [35] [Figure 1].

A recent systematic review analyzed the effects of vitamin C supplementation on glycemic control and cardiovascular risk factors in people with type 2 diabetes, suggesting that vitamin C supplementation may improve glycemic control and BP in people with T2DM, although larger, long-term and high-quality trials are still needed [36]. Furthermore, a number of observational studies have examined the associations of dietary and circulating vitamin C levels with MetS [37,38]. However, no final conclusion can be drawn. A recent meta-analysis of observational studies showed that both dietary and circulating vitamin C levels are inversely associated with MetS, although further well-designed prospective studies are needed [39]. Given this background, an extensive search of SCOPUS, PubMed, and CENTRAL was performed using the following string: ((vitamin C) or (ascorbic acid)) AND (systematic review [pt] or meta-analysis [pt]) and 2015:2025 [dp]). The search string retrieved 505 manuscripts. Hand-searching of principal generalist, human nutrition, and basic research journals was also carried out. Two authors (V.O. and D.M.) independently reviewed the titles, abstracts, and full texts of the retrieved articles to determine their potential inclusion. Any disagreements were resolved via discussion with a third author (S.C.). Manuscripts concerning the role of vitamin C in type 2 diabetes and metabolic syndrome were considered. Although the string appeared limited to systematic review and meta-analysis, the screening of 505 manuscripts produced a narrative review that summarizes and integrates the highest quality of evidence available on the topic, by pooling and evaluating the results of multiple primary studies such as observational studies and randomized clinical studies, offering a more robust conclusion than individual randomized clinical trials. This review aims to explore the pathophysiological role and therapeutic potential of vitamin C in T2DM and MetS.

## 2. Vitamin C Metabolism

**Figure 2:**
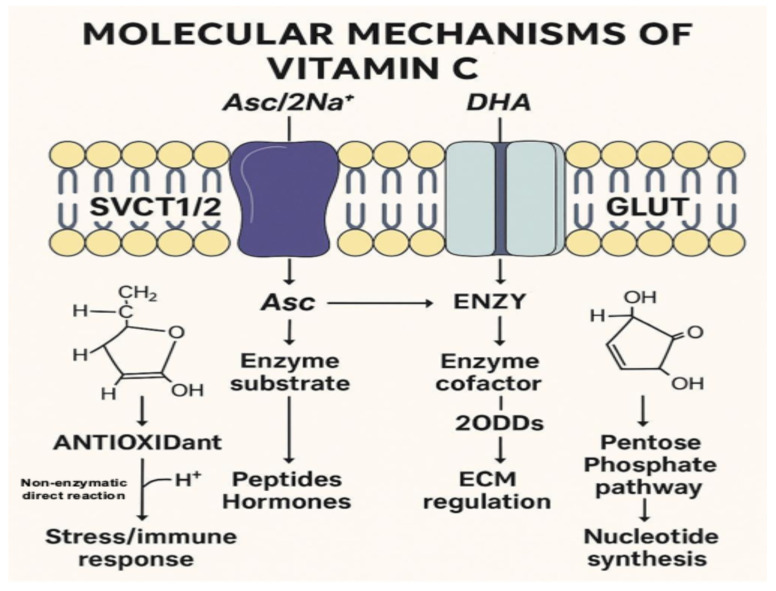
Molecular Mechanisms of Vitamin C. This infographic integrates the main molecular and biochemical roles of Vitamin C (ascorbate, Asc) within mammalian cells. The illustration depicts: Cellular Uptake: Vitamin C enters the cell in its reduced form (Asc) through sodium-dependent vitamin C transporters (SVCT1/2) and in its oxidized form (dehydroascorbate, DHA) through glucose transporters (GLUTs). Redox Cycling: Inside the cell, Asc and DHA interconvert, maintaining the redox balance essential for cellular metabolism. Antioxidant Function: Asc scavenges reactive oxygen species (ROS) through non-enzymatic antioxidation, protecting cells from oxidative stress and supporting immune and stress responses. Enzymatic Cofactor Role: Ascorbate acts as a cofactor for 2-oxoglutarate-dependent dioxygenases (2OGDDs), enzymes that regulate collagen biosynthesis, HIF-1α degradation, and epigenetic modifications. Biosynthetic Pathways: Asc participates in peptide and hormone synthesis and supports the pentose phosphate pathway, promoting nucleotide production and cellular energy homeostasis. The design merges the structural, transport, and enzymatic mechanisms found in biochemical and physiological contexts, suitable for inclusion in a scientific review or didactic article.

Ascorbic acid, commonly known as vitamin C, is a hydrophilic micronutrient essential to human health. While many plants and animals are capable of synthesizing it, humans must obtain it through dietary sources [40]. Vitamin C is a crucial micronutrient involved in numerous physiological processes in the human body. It is commonly found in various foods such as fruit and vegetables, dietary cereals, or as vitamin supplements in energy tabs [41]. The compound L-ascorbic acid, the active form of vitamin C, is essential for humans, as it cannot be synthesized endogenously, making dietary intake necessary. Vitamin C also neutralizes reactive oxygen species such as superoxide anions and inhibits the formation of peroxynitrite, contributing to the maintenance of vascular endothelial integrity, thereby contributing to improved vascular endothelial function [42,43]. Vitamin C, or ascorbic acid, is an essential water-soluble compound and a hydrophilic micronutrient that plays a vital role in human physiology. Most plants and animals can synthesize it endogenously unlike humans who are unable to synthesize it autonomously because of the deficiency of the enzyme L-gulonolactone oxidase. For this reason, it must necessarily be introduced via the diet. Vitamin C exists in vivo mainly in a reduced form, ASC synthesize it endogenously an oxidized form, DHA, of which the former is by far the predominant one [44]. The total capacity of vitamin C, particularly for dietary absorption and that which is available, is considered the combined pool of ASC and DHA, consequentially to efficient intracellular recycling of DHA to ASC by most cell types [45]. In general, there are three potential modes of membrane transport: passive diffusion, facilitated diffusion and active transport [46]. After ingestion, vitamin C is primarily absorbed in the small intestine through sodium-dependent active transporters, in particular the transporter SVCT1 (Sodium-Dependent Vitamin C Transporter 1), which mediates the absorption of ascorbate, the biologically active reduced form. In parallel, the oxidized form, dehydroascorbate (DHA), can be absorbed through transporters of the GLUT family (in particular GLUT1, GLUT3 and GLUT4), and then rapidly reduced to ascorbate inside cells via intracellular redox mechanisms [47]. SVCT1 is present in high-capacity epithelial tissues, such as the intestine and the kidney, and serves particularly for dietary absorption and renal reabsorption. SVCT2 is more widespread in tissues that require high intracellular concentrations of vitamin C, such as the brain, endocrine tissues and nerve cells [48,49]. Other studies showed that concentration of SVCT1 and SVCT2 varies along the intestinal tract: the colon, for example, has much lower levels of these transporters compared to the duodenum or jejunum, both at the mRNA and at the protein level, resulting in lower capacity for ascorbate absorption in that region [50]. Some organs, such as the brain, adrenal glands and leukocytes, have intracellular concentrations much higher than plasma, thanks to the activity of the transporter SVCT2, which is abundant in metabolically active tissues. These active transport systems allow for selective tissue accumulation even under suboptimal intake, suggesting a protective mechanism for tissues considered priority for survival and cellular function [51]. In particular, in the brain, vitamin C is transported into neuronal cells via SVCT2, which allows for intracellular accumulation against its concentration gradient, protecting the tissue from oxidative stress; this is important both for normal function and for response to damage or deficiency [52,53]. After absorption, vitamin C is distributed to the various tissues of the body. An important element is that this distribution is not uniform: some tissues, such as the brain and the adrenal glands, reach saturation (that is, the maximum acceptable/tolerated content) at relatively low levels of intake. Studies in animals—for example in guinea pigs, which do not synthesize vitamin C, as humans do—have shown how in different dietary regimens brain saturation occurs early, and that in conditions of deficiency the brain is able to maintain a significant fraction of its normal concentrations, while other tissues collapse significantly more [54]. Once in the intestine, ascorbate is absorbed, while dehydroascorbate (the oxidized form) can be transported via glucose transporters (GLUT 1, 3, 4) and then reduced again inside cells. However, under physiological conditions, the contribution of dehydroascorbate to the total pool is considered smaller than that of direct ascorbate, also because high concentrations of glucose in plasma can inhibit dehydroascorbate transport via the GLUTs [48]. In the brain, vitamin C is transported into neuronal cells via SVCT2, which allows intracellular accumulation against the gradient, protecting the tissue from oxidative stress; this is important both for normal function and for response to damage or deficiency [52,53]. The elimination of vitamin C occurs mainly by renal route. In renal glomeruli, ascorbate is freely filtered and subsequently reabsorbed in the proximal tubules, which are also equipped with SVCT1 transporters. However, reabsorption is subject to a maximum threshold: once the transport capacity is exceeded, the excess vitamin C is eliminated in the urine. This mechanism explains why increasing intake beyond saturation levels (~200 mg/day in healthy subjects) does not lead to a linear increase in plasma concentrations, but rather to greater urinary excretion [55]. Another crucial aspect is renal regulation. In the kidneys, ascorbate is filtered in the glomeruli and then reabsorbed in the proximal tubules via SVCT1. There is however a renal threshold beyond which the excess is eliminated in the urine. Recent studies in healthy subjects have estimated that this threshold—that is, the plasma concentration above which vitamin C appears in urine—is around 48–60 µM, with some difference between men and women [56]. Finally, it is important to note that pathological conditions such as diabetes mellitus, chronic kidney disease and certain genetic mutations can impair conservation of vitamin C, leading to urinary losses and functional deficiencies even in the presence of adequate intakes. In these contexts, regulation of the expression of SVCT transporters is correlated to adapting the efficiency of tissue and renal transport to systemic availability [47]. For example, in diabetes mellitus, a condition known as ‘renal leak’ may occur, where vitamin C is excessively excreted in urine despite plasma concentrations being below the normal excretion threshold, resulting in lower systemic levels; this leads to lower plasma levels compared to healthy subjects. Factors such as hyperglycemia, vascular complications, proteinuria seem to contribute to this phenomenon [56]. In the context of chronic kidney disease, especially in pediatric patients with advanced CKD stage or on dialysis, vitamin C deficiency is frequent. Even when a significant oral supplemental dose is administered (for example 250 mg/day), many patients do not reach “normal” serum levels, probably due to increased losses, impaired absorption, or altered distribution [57]. Finally, the stable plasma concentrations, at rest, in healthy subjects typically lie within a range that allows renal saturation without overload; the pharmacokinetics of vitamin C is highly dependent on dose—that is, the fraction absorbed, the rate of excretion and the distribution in tissues change much more than proportionally to intake when certain thresholds are exceeded [58]. Vitamin C exerts multiple biological activities that likely underlie its immunomodulatory properties [Figure 2]. Its potent antioxidant capacity stems from its readiness to donate electrons, which enables it to safeguard key biomolecules such as proteins, lipids, carbohydrates, and nucleic acids from oxidative damage generated during normal cellular metabolism and through exposure to toxins and pollutants (for example, cigarette smoke) [59]. Vitamin C serves as an essential cofactor for several biosynthetic monooxygenase and dioxygenase enzymes, as well as for enzymes involved in gene regulation [60]. Additionally, it is necessary for the activity of two hydroxylases involved in the biosynthesis of carnitine, a molecule that facilitates the mitochondrial transport of fatty acids for energy production, and also as a cofactor for lysyl and prolyl hydroxylases needed for stabilizing the tertiary structure of collagen [61]. Intracellular redox metabolism allows the oxidized (or partially oxidized) vitamin C to be regenerated via systems dependent on glutathione, NADPH and other enzymatic mechanisms, limiting the permanent loss of antioxidant activity. If dehydroascorbate is not regenerated, it can degrade into irreversible metabolites such as 2,3-diketo-gulonic acid, which may further decompose into products such as oxalate [62,63]. Recent discoveries highlighting the involvement of vitamin C in key biochemical pathways that regulate epigenetic mechanisms have renewed scientific interest in this molecule. These findings suggest that ascorbate contributes to cellular adaptation to toxins, pharmaceuticals, and various stressors through mechanisms that go beyond its classical antioxidant role.

## 3. Vitamin C and Insulin Resistance

Vitamin C is the most potent hydrophilic antioxidant with the ability to scavenge reactive oxygen species (ROS), nitrogen species and regenerate other antioxidants, such as vitamin E [55,56,57]. Oxidative stress is a major contributor to the pathophysiology of T2DM, leading to β-cell dysfunction, impaired insulin signaling, mitochondrial alterations, and insulin resistance [64,65]. Furthermore, chronic hyperglycemia interferes with cellular uptake of vitamin C, leading to relative intracellular deficiency [66]. Experimental studies have further demonstrated that vitamin C supplementation improves skeletal muscle insulin sensitivity and ameliorates mitochondrial dysfunction, suggesting a plausible mechanistic link to improved glycemic control [67].

In this context, there has been growing scientific interest in using nutritional supplements—including probiotics, soluble fibers, resveratrol, as well as various vitamins and minerals—to help manage metabolic disorders. In particular, multiple recent systematic reviews and meta-analyses have evaluated the impact of vitamin C supplementation in individuals with type 2 diabetes.

Khodaeian M et al. [68] conducted a meta-analysis including three RCTs that evaluated vitamin C supplementation in T2DM patients, with daily doses ranging from 800 to 1000 mg administered orally for 4 to 16 weeks. The pooled results showed that Vitamin C supplementation fails to produce a meaningful reduction in insulin resistance as measured by the Homeostasis Model Assessment (HOMA) index (standardized mean difference: −0.150; 95% CI: −0.494 to 0.194; *p* = 0.391) either alone or in combination with other antioxidants. Several limitations may account for the lack of observed benefit. First, the number of trials assessing vitamin C supplementation alone was limited, with relatively small sample sizes. Second, heterogeneity in dosage, trial duration, and baseline patient characteristics complicates interpretation. Third, impaired vitamin C transport in hyperglycemic states may reduce intracellular availability, potentially attenuating therapeutic efficacy. Finally, the dual antioxidant/pro-oxidant behavior of vitamin C, influenced by dose and metabolic context, may confound outcomes in clinical settings. Conversely, Fong et al. [69] demonstrated that vitamin C supplementation may improve indices of glycemic control. In particular, they reported significant reductions in fasting blood glucose (FBG: MD −0.74 mmol/L), glycated hemoglobin (HbA1c: MD −0.54%), and postprandial glucose (MD −0.95 mmol/L), without any side effects in the population taking vitamin C supplementation. These effects were more evident in trials of >12 weeks duration and in individuals with elevated baseline HbA1c, suggesting greater benefit in poorly controlled patients. However, these findings are affected by the low certainty of the evidence, the variability of the period and the dose of administration. Furthermore, at higher doses vitamin C determined an increase in FBG values. No significant data were found relating to insulin resistance (such as fasting insulin, HOMA-IR, or clamp-derived insulin sensitivity).

Later, Chai MSc Y et al. [70] performed an umbrella review to reassess the existing systematic reviews and meta-analyses (SRMAs) investigating the effects of water-soluble vitamin supplementation on glycemic control for patients with T2DM. The evidence consistently indicates significant reductions in fasting blood glucose (FBG) particularly in trials exceeding 30 days of supplementation and, even with some discrepancies, in glycated hemoglobin (HbA1c), likely due to altered bioavailability of vitamin C which depends from transport proteins, that are impaired in T2DM. Conversely, pooled analyses evaluating the effects on insulin resistance indices (HOMA-IR, fasting insulin concentrations) reported inconclusive or non-significant results. This discrepancy suggests that while vitamin C may reliably improve glycemic indices, its influence on insulin resistance remains uncertain. Unfortunately, the strength of the available evidence is limited by several methodological shortcomings, such as the low or very low quality of meta-analyses (according to AMSTAR-2 and GRADE assessments), the high heterogeneity, risk of bias, and inadequate reporting of study quality. Also, the wide variability in dosing (72–6000 mg/day) and intervention duration (2 weeks to 9 years) complicates interpretation and clinical translation. Future randomized controlled trials should be adequately powered, employ standardized dosing regimens, and extend intervention duration to capture both immediate and long-time frames effects on glycemic regulation and insulin resistance. Investigations should also address the pharmacokinetics and tissue bioavailability of vitamin C in diabetic populations.

## 4. Vitamin C, Diabetes and Its Complications

### 4.1. Vitamin C and Diabetes

The global incidence of diabetes has reached epidemic proportions. Globally, approximately 589 million adults (20–79 years) are affected by diabetes, and this figure is expected to reach 853 million by 2050 [71]. People living with diabetes face a greater likelihood of illness and premature death. Chronic hyperglycemia, together with episodes of hypoglycemia, contribute to the development of diabetes-related complications such as retinopathy, neuropathies, nephropathy, myocardial infarction, coronary heart disease, cerebrovascular disease, cardiomyopathy and peripheral artery disease. These complications may result in serious disability and premature mortality. Furthermore, gestational diabetes raises the likelihood of delivering infants with macrosomia and heightens the mother’s risk of manifesting type 2 diabetes and cardiometabolic disease later in life, while also predisposing offspring to impaired glucose metabolism [72,73].

### 4.2. Pathophysiology of Oxidative Stress in Diabetes

Oxidative stress is characterized by an imbalance between oxidants and antioxidants, which may culminate in molecular injury [74]. Oxidants comprise reactive oxygen species and reactive nitrogen species. These reactive molecules can be produced intracellularly, extracellularly, or within cellular organelles. Their production occurs through mitochondrial activity during aerobic metabolism, as well as via enzymatic pathways involving uncoupled nitric oxide synthase (NOS), nicotinamide adenine dinucleotide phosphate oxidases (NADPH), xanthine oxidase, lipoxygenases and cytochrome P450 monooxygenases. By inducing reversible post-translational protein modifications, certain oxidants modulate cell signaling, gene expression, and various biological processes [75]. While, for many biological processes, redox signaling and oxidant production are essential, excessive oxidants—beyond the capacity of non-enzymatic (e.g., vitamins E and C, glutathione) and enzymatic antioxidants (e.g., glutathione peroxidases, SODs, catalase)—can cause DNA and lipids damage and irreversible modifications of proteins. Such oxidative stress has been implicated in the development of chronic diseases, including cardiovascular disease, neurodegenerative disorders and cancers has been implicated [76,77,78].

Disturbances in nitrosative and oxidative redox homeostasis play a pivotal role in the pathogenesis of type 2, type 1, and gestational diabetes, as well as their associated complications [79]. In fact, patients exhibiting hyperglycemia show markedly increased levels of oxidative stress markers [80]. Elevated glucose levels promote oxidative stress and the generation of advanced glycation end products via irreversible, non-enzymatic protein modifications. These alterations impair pancreatic β-cell function, decrease insulin sensitivity, and cause cellular and tissue damage, thereby driving the progression of diabetes complications [79]. Consistent findings from multiple observational studies suggest that individuals with diabetes generally present lower vitamin C levels than healthy subjects [81,82,83]. Even at the prediabetic stage, this reduction can be observed and appears to reflect the influence of higher body weight and associated inflammatory markers (e.g., C-reactive protein), rather than inadequate dietary intake of vitamin C [83].

In the next sections, we review the effects of supplementation of vitamin C on various outcomes related to diabetes.

### 4.3. Possible Role in Diabetes Mellitus Prevention

The potential role of vitamin C in the prevention of type 2 diabetes mellitus (T2DM), has been extensively investigated. Lampousi et al., in their recent systematic review and meta-analysis, which included 25 prospective observational studies and 15 randomized controlled trials (RCTs), evaluated the association between T2DM risk, insulin resistance, and β-cell functions, as well as supplementation, circulating levels and dietary intake of vitamin C. This study highlighted that, between dietary vitamin C and type 2 diabetes, there was a nonlinear dose–response relationship; an intake of 70 mg/day was linked with a 24% lower risk of type 2 diabetes (RR: 0.76; 95% CI: 0.61, 0.95), whereas a further reduction in risk was not associated with higher intakes. Subgroup analyses revealed that smoking may reduce vitamin C’s protective effects because of depleted circulation of vitamin C and increased oxidative stress in smokers [84]. Nonetheless, for the primary prevention of type 2 diabetes, randomized controlled trials provide no robust evidence supporting the use of vitamin C supplementation [79]. An inverse association was found between plasma vitamin C levels and development of islet autoimmunity in children with elevated genetic susceptibility, suggesting vitamin C levels may influence the risk of type 1 diabetes [85]. An altered ratio of oxidized to reduced vitamin C, resulting from oxidative stress or inefficient vitamin C recycling, may increase the risk of type 1 diabetes by negatively affecting beta cell function [86]. Additional observational studies found no link between dietary vitamin C consumption and the likelihood of developing type 1 diabetes in a prospective case–control study conducted in Sweden [79,87]. As far as the authors are aware, no randomized controlled trials have evaluated the impact of vitamin C supplementation on type 1 diabetes prevention. Considering the inconsistent results from human studies, it is possible that vitamin C levels may instead indicate higher consumption of other protective compounds—especially those present in fruits and vegetables—rather than a direct effect of vitamin C itself. Furthermore, because more pronounced correlations are seen in cross-sectional studies than in prospective ones, the reduced vitamin C levels noted in people with diabetes may result from the disease itself instead of serving as a causal factor in its onset [79].

### 4.4. Impact of Vitamin C Supplementation on Glycemic Regulation

Vitamin C, because of its well-known antioxidant effects, has been widely studied as a potential adjunct for enhancing glycemic control, given that oxidative stress is a major contributor to the development and advancement of type 2 diabetes and its complications. In this context, Ashor et al. reviewed and meta-analyzed 22 RCTs with 937 adult participants to assess how vitamin C supplementation influences insulin, blood glucose and HbA1c levels. Overall, the analysis showed that vitamin C supplementation has no significant effect on HbA1c, insulin and blood glucose levels in the general population. However, subgroup analyses revealed that in subjects with T2DM, vitamin C supplementation led to a statistically significant reduction in fasting plasma glucose, particularly in interventions lasting more than 30 days. Moreover, a beneficial effect on fasting insulin levels was noted, whereas no notable changes were detected in postprandial insulin concentrations. Meta-regression analyses identified several clinical variables that modulated the response to supplementation: older age, higher BMI, elevated baseline glucose levels, and longer intervention duration were all associated with greater improvements. In contrast, no associations were detected with dosage or baseline plasma vitamin C concentrations. Interestingly, no significant effect was observed on HbA1c, suggesting that vitamin C may predominantly influence short-term glycemic markers rather than long-term glycemic control [33].

Nosratabadi et al. recently conducted a systematic review and meta-analysis of 22 RCTs, involving 1447 patients with T2DM, to clarify the role of vitamin C in modulating key glycemic indices. Pooled analysis indicated that administration of vitamin C was correlated with reductions in fasting blood glucose (FBG), fasting insulin concentrations, and HbA1c relative to untreated control groups. Notably, the decrease in HbA1c was more pronounced in studies with an intervention duration of ≥12 weeks and daily doses of ≥1000 mg of vitamin C. Conversely, no overall significant effect was observed on insulin resistance (HOMA-IR). However, subgroup analyses revealed that high-dose supplementation (≥1000 mg/day) produced a significant improvement in HOMA-IR. These results indicate that prolonged, high-dose vitamin C administration may contribute to an overall improvement in the glycemic profile of patients with T2DM. Nevertheless, the authors highlight the substantial heterogeneity among the included studies and emphasize the need for large, high-quality clinical trials to validate these findings and determine the most effective dosage and treatment period [88]. Other meta-analyses of RCTs have shown that vitamin C supplementation significantly reduces fasting blood glucose, HbA1c, and postprandial glycemic levels in type 2 diabetes patients, particularly with doses of 500–1000 mg/day, treatment durations of ≥12 weeks, and in patients with higher baseline HbA1c [79].

### 4.5. Effect of Supplementation of Vitamin C in Gestational Diabetes

Reports have shown an amplified incidence of oxidative stress biomarkers, like lipid peroxidation products and markers of DNA damage, in the blood of women diagnosed with Gestational Diabetes Mellitus (GDM) [89,90,91]. On the basis of these findings’ antioxidants might have a potential role in moderating GDM’s risk, so as part of a GDM prevention approach, it is important to pay attention towards dietary sources of antioxidants. Vitamin C is a nutritional antioxidant and its therapeutic potential in T2DM has been well-established in this review and in the other literature, with studies validating its beneficial effects on glucose metabolism and insulin sensitivity [36,91].

Despite these findings, evidence linking vitamin C intake to GDM risk remains poor, and the existing studies account for inconsistent results. A comprehensive meta-analysis of observational studies explored this association, providing valuable insights into the contribution of vitamin C to maternal and neonatal health. The analysis included 15 studies from different regions (13 case–control and 2 cohort designs), encompassing a total of 10,131 women of reproductive age with varying levels of vitamin C exposure, where the primary outcome was the diagnosis of GDM.

Four of the included studies identified a relationship between vitamin C exposure and GDM incidence, with two evaluating both dietary intake and blood concentrations. Overall, women with lower vitamin C exposure showed a greater risk of developing GDM, and three studies reported statistically significant results. Subgroup analyses considered factors such as exposure type, duration, geographic region, and diagnostic criteria. Findings revealed that lower internal vitamin C concentrations—particularly in populations from India, Europe, and America, and during the second or third trimester—were associated with a higher risk of GDM according to the American Diabetes Association (ADA) diagnostic standards.

The meta-analysis examined both internal (blood concentration) and external (dietary intake) exposure scenarios. Internal exposure was regarded as a more comprehensive measure, as it reflects cumulative vitamin C intake from multiple sources and minimizes variability in absorption and metabolism. However, several limitations were identified. First, the studies demonstrated considerable heterogeneity, stemming from differences in diagnostic criteria, exposure timing, and measurement methods. For example, while the International Association of Diabetes and Pregnancy Study Groups (IADPSG) defines GDM based on exceeding a single threshold in the oral glucose tolerance test, the ADA requires two or more thresholds to be surpassed [91,92]. Studies have shown that the GDM diagnosis increased under the International Association of Diabetes and Pregnancy Study Groups standard [91,93]. This suggests that women diagnosed according to ADA guidelines might experience more pronounced oxidative stress and thus lower circulating vitamin C levels [91]. A second limitation concerns the absence of randomized controlled trials (RCTs) directly evaluating the relationship between vitamin C and GDM, as well as the small number of large-scale cohort studies. Most available data come from case–control studies, which provide a lower level of evidence. Third, several investigations on external vitamin C exposure overlooked supplementation, focusing solely on dietary intake, which may have influenced results. Additionally, factors such as genetic predisposition, nutrient–nutrient interactions, and bioavailability could affect how vitamin C influences disease risk.

In summary, although vitamin C shows potential in reducing the likelihood of GDM, it should be regarded as one component of a broader, integrative approach to prenatal health [91,94].

Consequently, promoting the consumption of vitamin C–rich foods and maintaining adequate blood concentrations during pregnancy may represent beneficial preventive measures against GDM [91].

### 4.6. Impact of Vitamin C Intake on Diabetic Complications

Chronic renal disease is increasingly common, largely due to obesity, diabetes, and hypertension. As kidney function declines, the body faces both greater needs for water-soluble vitamins and greater losses of these nutrients, especially during dialysis. This makes the role of supplementation an important part of patient care [95]. In the early stages of CKD, supplementation is generally unnecessary, provided patients follow a balanced diet. As the disease progresses, however, deficiencies become more likely. At this point, vitamins such as folate and B12 can help lower homocysteine levels, which are linked to cardiovascular risk. From stage 3b onwards, niacin is often useful to reduce phosphate levels, while thiamine becomes critical because it is heavily lost during dialysis and protects against neurological complications [96,97].

Vitamin C deserves special mention. It supports iron mobilization, reduces inflammation, and can improve anemia in dialysis patients. Yet, while moderate doses are beneficial, high doses raise the risk of oxalate accumulation and kidney stones. Vitamin B6 also plays a role in bone and blood health, but excess supplementation may interfere with anemia treatments [98,99,100].

Nutrition itself is fundamental. Adherence to a Mediterranean diet, emphasizing vegetables, legumes, fish, and olive oil with reduced red meat intake, has demonstrated positive effects on patient health. In contrast, a Western diet heavy in processed foods, sugary drinks, and red meat worsens disease progression. Vegetarian diets can also be beneficial but must be balanced to avoid protein malnutrition [101]. Antioxidant vitamins such as B, C, and E play a role in lowering oxidative stress, which is a significant driver of CKD advancement. Evidence shows some benefit, particularly in dialysis patients, though results are mixed in earlier stages. Another emerging area of interest is the gut microbiota, since CKD alters intestinal bacteria in ways that affect vitamin metabolism and increase toxin accumulation. Pre- and probiotics may therefore offer supportive benefits.

In conclusion, the need for vitamin supplementation in CKD depends on disease stage. Early CKD can often be managed with a nutrient-rich diet alone. Advanced CKD and dialysis usually require supplementation with folate, B12, B6, niacin, thiamine, and vitamin C. However, high intake levels should be approached with caution, since they can negatively affect heart performance or raise the risk of oxalate accumulation. Overall, combining a nutrient-rich, plant-based diet with specific supplements appears to be the most beneficial strategy for delaying CKD progression and improving life quality [102,103].

Diabetic foot ulcers are among the most severe complications of diabetes, responsible for high treatment costs, frequent amputations, and increased mortality. Their healing is often delayed because of impaired immune responses, vascular damage, and nutritional deficiencies. Micronutrient status plays a crucial role in this context. Deficiencies in essential micronutrients adversely affect tissue repair processes by impairing collagen synthesis, prolonging the inflammatory phase of wound healing, and weakening immune competence. In particular, reduced serum levels of vitamin D, magnesium, selenium, and vitamin C are frequently reported in individuals with diabetic foot ulcers. These deficits are associated with slower healing, higher infection risk, and worse clinical outcomes [104,105]. Vitamin C and selenium also support antioxidant defenses and collagen synthesis, both vital for wound healing [105]. While factors such as age, duration of diabetes, and vascular complications cannot be changed, nutritional status is modifiable. Regular monitoring of micronutrient levels, coupled with targeted supplementation and personalized nutrition strategies, can therefore play an important role in managing diabetic foot ulcers and improving healing outcomes [104]. Among the major complications of diabetes, diabetic retinopathy (DR) frequently remains undetected and continues to represent one of the primary causes of vision impairment in the working-age population. While glycemic control and blood pressure management remain fundamental in diabetes care, growing evidence underscores the significant role of diet and lifestyle in the development and progression of diabetic retinopathy (DR) [106]. Systematic reviews have consistently shown that adherence to a Mediterranean-style diet—emphasizing fruits, vegetables, legumes, fish, olive oil, and nuts—provides a protective effect against DR. Adequate intake of fiber, oleic acid, and tea is also associated with lower risk. In contrast, diets high in calories, rice, choline, diet soda, sugar, and sodium have been linked to higher incidence or faster progression of DR and diabetic macular edema (DME). To translate this evidence into practice, researchers have proposed a food pyramid for eye health [107] in which at its base there are foods rich of rich in folic acid, vitamin C, and carotenoids such as lutein and zeaxanthin, vitamin E, polyphenols and antioxidant content. At the top of the pyramid, guidelines advise avoiding excess salt and sugar, while also recognizing that personalized supplementation (e.g., omega-3s, L-methylfolate, B vitamins, lutein/zeaxanthin) may be helpful if dietary intake is insufficient [108].

Importantly, physical activity complements nutrition, with regular aerobic and resistance exercise (30–40 min, several times per week) shown to support overall eye health and metabolic balance [109,110]. In conclusion, current evidence suggests that dietary counseling for diabetes should explicitly address eye health, not just glucose control. A nutrient-dense, predominantly plant-based Mediterranean diet, together with appropriate supplementation and consistent physical activity, represents the most effective approach to prevent or slow the progression of diabetic retinopathy and to preserve visual function in individuals with diabetes.

## 5. Vitamin C and Metabolic Syndrome (Mets)

MetS is characterized by a cluster of interconnected metabolic abnormalities (at least three of five) such as central adiposity defined by elevated waist circumference, arterial hypertension, dyslipidemia (elevated triglyceride (TG) levels and reduced high-density lipoprotein cholesterol (HDL-C)), and elevated fasting blood glucose levels (>126 mg/dL), which substantially increase the probability of developing T2DM and cardiovascular events [111]. As in T2DM, oxidative stress and low-grade systemic inflammation are recognized as pivotal mechanisms underpinning MetS pathophysiology. Given its role as a potent antioxidant and modulator of endothelial and immune function, vitamin C has been hypothesized to exert a protective effect against MetS.

Preclinical studies in animal models demonstrated that vitamin C supplementation reduced body weight, improved lipid and glucose profiles [including insulin resistance as evidenced by HOMA-IR], decreased blood pressure, and attenuated oxidative stress markers such as malondialdehyde (MDA) alone [112,113] or in combination with other antioxidants [114]. However, the interventions combined vitamin C with other antioxidants, making it more challenging to determine whether the observed effects can be attributed solely to vitamin C. Human observational studies consistently report lower vitamin C intake and circulating concentrations in individuals with MetS compared to controls [115]. Cross-sectional evidence suggests an inverse association between dietary vitamin C or plasma levels and MetS prevalence, as well as a protective effect against individual components such as waist circumference, triglycerides, and HDL-C [116,117,118]. Nevertheless, discrepancies exist, with some studies failing to detect significant differences in vitamin C status between MetS and non-MetS groups [119,120]. Interventional trials indicate that vitamin C supplementation, either alone or in combination with exercise or other antioxidants, may improve MetS-related parameters including BMI, triglycerides, LDL-C, and blood pressure [121,122]. However, the SU.VI.MAX study showed no preventive effect of long-term combined antioxidant supplementation on MetS incidence, though higher baseline serum vitamin C was associated with reduced risk [123].

In order to address these issues, Guo H et al. [39] conducted a comprehensive meta-analysis encompassing 28 observational studies with a cumulative sample exceeding 110,000 participants assessing both dietary intake and circulating concentrations of vitamin C in relation to MetS prevalence. They found that, among individuals with MetS, there was both a lower dietary of vitamin C levels compared with controls (SMD = −0.04; 95% CI: −0.08 to −0.01) and reduced circulating levels of vitamin C relative to controls (SMD = −0.82; 95% CI: −1.24 to −0.40), and both of which were negatively associated with MetS. As anticipated, oxidative stress, characterized by a disruption between the generation and neutralization of ROS, represents a defining feature of MetS. Overall, it is worsened by both the excessive intake of fat and carbohydrates together with low physical activity, as well as excessive production of pro-inflammatory mediators (which in turn stimulate macrophages and monocytes) by the adipose tissues. In healthy patients, the synthesis of ROS and the levels of natural antioxidant are equivalent. When ROS production increases, it causes lipid peroxidation. In this context, the underlying mechanism behind the negative link between vitamin C and MetS may be may stem from vitamin C’s ability to neutralize reactive free radicals by donating electrons, forming the stable ascorbyl radical and thereby reducing oxidative stress [124]. This action prevents oxidative damage to cellular components and has been associated with improvements in vascular function, including reductions in blood pressure through effects on cGMP-dependent protein kinase activity. Literature data showed that higher antioxidant vitamin intake improves total antioxidant status [125] and correlates with reduced waist circumference and favorable lipid profiles, as well as potential benefits in T2DM. In addition, another characteristic of MetS is chronic low-grade inflammation stimulated both by oxidative stress and adipocyte hypertrophy. It is characterized by the activation of inflammatory signaling networks, leading to adipokine imbalance and excessive systemic release of cytokines and chemokines. Accordingly, vitamin C has been shown to alleviate inflammatory responses in animal models [126] while human studies indicate its potential to attenuate inflammation in MetS [122,127]. Nevertheless, vitamin C plays an important role in modulating inflammation via effects on neutrophil function [128]. Since adipose tissue hypertrophy and hyperplasia contribute to the chronic inflammatory state in MetS, efficient neutrophil chemotaxis, phagocytosis, and clearance are critical. Vitamin C has been shown in both human and animal studies to enhance neutrophil chemotaxis, improve bacterial phagocytosis, and promote the clearance of apoptotic neutrophils by macrophages.

### 5.1. Vitamin C and Arterial Hypertension

Some studies also investigate the effects of micronutrients on blood pressure (BP) in patients with T2DM. About 25% of adults worldwide suffer from hypertension, and this percentage is expected to increase to 29% by 2025; furthermore, among hypertensive patients, more than 90% suffer from essential hypertension [129]. Hypertension represents a major risk factor for chronic diabetic complications, and about 71% of patients with T2DM in the United States have hypertension. Indeed, optimal BP levels are not achieved by most hypertensive type 2 diabetic patients [130]. Endothelial cell dysfunction is a consequence of damage to endothelial cells and the release of large amounts of superoxide anions [131]; it follows that it is connected to vascular disease and it has been shown that it plays a role in promoting hypertension and cardiovascular and cerebrovascular disease. In this field, vitamin C might improve endothelial-dependent vasodilation, by increasing intracellular concentrations of tetrahydrobiopterin, an endothelial nitric oxide synthase cofactor important in the production of nitric oxide in patients with essential hypertension and by reversing the effect of the nitric oxide synthase inhibitor NG-monomethyl-L-arginin [129].

According to a systematic review and meta-analysis, vitamin C supplementation (500 mg to 1500 mg/day) reduced diastolic BP (DBP) with a reduction of −2.88 mmHg, but not systolic BP (SBP) in T2DM patients [130]. Another interesting systematic review and meta-analysis evaluated SBP and DBP before and after vitamin C supplementation (at the dose >200 mg) and compared them between the intervention and control groups; the authors found a significant change in SBP and DBP. In the analysis of the subgroup of patients aged ≥60 years, there was a significant difference in SBP and DBP after vitamin C supplementation in comparison to those before supplementation and this difference is likely to be a consequence of the increase in hypertension with age. Furthermore, in this subgroup, observation and control groups showed significant differences in SBP and DBP [129]. The authors of another interesting systematic review and meta-analysis showed the hypotensive effect of vitamin C on SBP and DBP in T2DM patients: in vitamin C supplementation group they found a reduction in the proportion of patients above 130/80 mmHg from 88.8% to 42.1% after 12 months, while in placebo group they found a reduction from 92.1% to 83.5% after supplementation. However, vitamin C supplementation was considered to have a moderate certainty of reduction statistically and clinically on SBP (mean decreases in SBP was of 26.27 mmHg), while vitamin C supplementation was considered to have a very low certainty of reduction statistically and clinically on DBP (mean decrease in DBP was of 23.77 mmHg) [36].

### 5.2. Vitamin C and Lipid Profile

Vitamin C is a main factor in lipid modulation, as it is involved in the regulation of cholesterol catabolism to bile acids [132]. Indeed, it contributes to a reduction in LDL blood concentrations through conversion of cholesterol to bile acids and increasing LDL receptors on hepatocytes [132,133]. Moreover, vitamin C inhibits LDL oxidation and facilitates its uptake by LDL receptors on hepatocytes through its antioxidative effects [132,134].

A meta-analysis estimated vitamin C supplementation effects on lipid profile in diabetic patients (patients with T2DM). It significantly improved lipid profile by reducing triglycerides (TG) and total cholesterol (TC), but showed no overall significant change in LDL and HDL concentrations. Subgroup analysis showed that patients who received vitamin C in long-term (≥12 weeks) interventions were more likely to benefit than patients who receive short-term (<12 weeks) interventions. Moreover, it was documented a significant decrease in TG when patients received higher (>200 mg/day) versus lower doses (≤200 mg/day). Vitamin C supplementation significantly decreased serum LDL levels when administered for at least 12 weeks, but it failed to increase serum HDL levels. Thus, lipid profile improvement was influenced by the duration of vitamin C treatment and dose. Additional high-quality and long-term RCTs conducted in individuals with different serum concentrations and dietary intakes of vitamin C are needed to confirm these results [132].

Conversely, a 12-month clinical trial showed different results. The study involved 456 diabetic patients who were randomly assigned to three groups: control arm (metformin + placebo), parallel arm I (metformin + ascorbic acid), and parallel arm II (metformin + acetylsalicylic acid). A significant reduction in LDL-c, TG, and Total Cholesterol (TC) was shown in the ascorbic acid group compared to the control group.

In a Systematic Review of 2022, 21 RCTs were selected. The study included RCTs about vitamin C’s therapeutic effects, compared to placebo, on outcomes related to CVD in diabetic patients or with MetS. The primary outcomes contained basic metabolic profiles such as blood glucose levels, and CVD-risk measurements like lipid profiles, endothelial function, and blood pressure. The secondary outcome was biomarkers of inflammation and oxidative stress.

However, the beneficial effects of vitamin C intake on reducing cholesterol levels and improving metabolic function in patients with diabetes or metabolic syndrome were reported in overwhelming. The reduction in CT was associated with amelioration of endothelial dysfunction and attenuation of oxidative stress [19,67,135,136,137].

Some studies showed that a dose of 1000 mg of vitamin C daily for 2–4 weeks does not significantly affect BP, nor triglycerides, HDL, and LDL values [137,138,139], even when taken for a prolonged period (4 years) at a lower dose of 275 or 133 mg daily [137,138]. In others, vitamin C at 800–1000 mg for 2–4 weeks also does not influence inflammatory cytokines, FPG or insulin levels in diabetic patients. Summarized, most antioxidant therapies have been dismissed in clinical trials, and this highlights the need for additional RCTs to confirm these effects, especially for patients with T1D.

Future clinical studies should aim to include larger populations and longer intervention periods, with particular attention to stratifying participants according to their initial glycemic status, in order better to confirm the potential benefits of vitamin C supplementation. Moreover, future research efforts should incorporate qualitative meta-analytical approaches to reinforce the current evidence base. This recommendation arises from one of the major limitations observed in the previously reviewed studies, where the significant heterogeneity among included randomized controlled trials (RCTs) may have influenced the reliability and interpretation of their findings [137].

### 5.3. Vitamin C and Obesity

Numerous studies have reported alterations in immune cell populations among individuals with obesity. There is a shift in macrophage polarization from the anti-inflammatory M2 phenotype toward the pro-inflammatory M1 type. This immunological imbalance is closely linked to hallmark features of obesity, including chronic low-grade inflammation and insulin resistance.

Additionally, obese subjects often display elevated levels of inflammatory mediators, such as tumor necrosis factor-alpha (TNF-α)—a key cytokine in obesity-related inflammation—which activates intracellular signaling cascades like IκB kinase (IKKβ) and c-Jun N-terminal kinase (JNK), both of which contribute to insulin resistance. Increased concentrations of other inflammatory markers, including interleukins (IL-6, IL-4), leptin, and macrophage chemoattractant protein-1 (MCP-1), have also been observed. A positive correlation has been established between the degree of obesity and the levels of these inflammatory biomarkers.

ROS play a crucial role in the onset and progression of obesity and its related metabolic disturbances. Consequently, antioxidant compounds such as carotenoids, vitamins E and C, zinc, magnesium, and selenium may exert protective effects against obesity-related complications [140,141,142,143,144].

Several mechanisms have been proposed to explain the association between vitamin C and anthropometric parameters. As a potent antioxidant, vitamin C neutralizes free radicals, thereby lowering oxidative stress and helping prevent obesity and its complications. Furthermore, vitamin C mitigates hypoxia within expanding adipose tissue—a process that contributes to obesity—due to its protective activity against ROS. The vitamin also modulates the activity of antioxidant enzymes such as paraoxonases (PONs) and peroxiredoxins (PRDXs), both of which are involved in defense mechanisms against oxidative damage.

In addition, vitamin C appears to influence adipocyte differentiation, inhibiting the maturation of fat cells by suppressing glycerol phosphate dehydrogenase (GPDH) activity. Acting as a competitive inhibitor of adenylate cyclase, vitamin C also decreases intracellular cyclic adenosine monophosphate (cAMP) levels—an important factor in adipogenesis.

A considerable number of studies have explored the relationship between serum or dietary vitamin C status and body composition indicators across different populations. However, their findings are often inconsistent. Some investigations reported no significant differences in vitamin C concentrations among normal-weight, overweight, and obese subjects [145,146], while others reported meaningful inverse associations [144,147,148]

A systematic review and meta-analysis including 84 observational studies (cross-sectional, case–control, and cohort designs) evaluated adults over 18 years without restrictions regarding ethnicity, sex, or publication date. The analysis examined associations between dietary intake or circulating vitamin C levels and anthropometric outcomes. Twelve of these studies found a significant inverse correlation between BMI and serum vitamin C (r = −0.17, 95% CI: −0.25, −0.09, I^2^ = 72.8%), indicating that higher vitamin C intake was associated with lower BMI [144,149]. Seventeen studies reported notably lower vitamin C levels in obese individuals compared with normal-weight participants (MD = 7.03, 95% CI: 3.80–10.26, I^2^ = 91.7%), whereas no significant difference was observed between normal-weight and overweight groups (MD = 0.60 μmol/L, 95% CI: −3.57–4.87, I^2^ = 94.6%).

Although some studies failed to identify associations between BMI or waist circumference (WC) and serum vitamin C, most reported inverse relationships between these anthropometric measures and vitamin C concentrations. Based on these findings, individuals with higher body mass appear to require greater vitamin C intake to maintain adequate serum levels. An additional 10 mg of vitamin C per day for every 10 kg increase in body weight has been suggested to achieve optimal health benefits while minimizing deficiency or toxicity risks. Most health authorities recommend a maximum daily intake of 1–2 g. The primary sources of vitamin C are fresh fruits and vegetables, whereas eggs, meat, grains, and dairy contain only minimal amounts [144]. A 12-month single-blind multicenter randomized controlled trial also compared the effects of combining metformin with ascorbic acid or acetylsalicylic acid on cardiovascular complications in patients with type II diabetes mellitus. Participants were divided into three groups: a control arm (metformin + placebo), parallel arm I (metformin + ascorbic acid), and parallel arm II (metformin + acetylsalicylic acid). The study aimed to evaluate the potential of these agents to enhance glycemic control and reduce diabetes-related cardiovascular risk. However, neither ascorbic acid nor acetylsalicylic acid significantly affected anthropometric parameters, either within or between groups [150]. The variability observed across these studies may stem from differences in sample size, genetic background, geographic location, and nutritional status of participants, all of which can influence outcomes and explain the inconsistencies in the existing literature.

## 6. Vitamin C and Cardiovascular Diseases

It is well known that among the chronic complications of T2DM there are cardiovascular events and, in this field, diet can influence the risk of ischemic heart diseases and in the United States it also accounts for about 45% of all cardiometabolic deaths in adults [150,151]. Indeed, the American Heart Association in its latest scientific statement recommends Mediterranean diet and DASH (the Dietary Approach to Stop Hypertension), that represents dietary patterns with little quantity of some nutrients like saturated fat and sodium, generally associated with higher Cardiovascular Disease (CVD) risk, and with high quantity of other micronutrients, like phytochemicals, unsaturated fatty acids, antioxidant vitamins and minerals, for the purpose of preventing or treating CVDs and T2DM [152]. According to data available, about 52% of the population takes supplements and in detail 31% of the population takes multivitamins, 19% takes vitamin D, 14% takes calcium, and 12% takes vitamin C; furthermore, annual American spending on dietary supplements is more than $30 billion. However, no general agreement exists about indication of taking individual vitamins and minerals or their combinations in order to prevent or treat CVD, but a good diet as part of a healthy lifestyle is generally and internationally recommended [153]. A healthy diet is composed of micronutrients, of which vitamin C is an essential component because it works as an antioxidant agent by eliminating free radicals, reduces inflammatory and platelet activity, while maintaining the homeostasis of endothelial cells and cardiac function and it represents a cofactor for many important metabolic processes and as a non-enzymatic quencher for free radicals. In T2DM patients there is an exposure to oxidative stress as a consequence of increased Reactive Oxygen Species (ROS) production and lipid peroxidation and in this context oxidative stress plays a pathophysiological role in causing CVD [151,152]. However, the studies currently available regarding the association between vitamin C and CVD show conflicting results and a possible explanation could be the differentiation in the oxidation of C-vitamers, vitamin C, and other common ascorbic acid derivatives used as supplements, so the beneficial effect of vitamin C may be different, and sometimes opposite, based on the fact that intake is from natural sources or from supplements, indeed fresh fruit and vegetables should probably be preferred compared to vitamin C supplementation in T2DM patients [151].

An interesting recent systematic review and meta-analyses evaluated 27 types of micronutrient involving 884 randomized controlled interventions for a total of 883.627 participants and it found moderate- to high-quality evidence in the reduction in CVD risk factors after supplementation with n-3 fatty acid, n-6 fatty acid, L-arginine, melatonin, folic acid, magnesium, flavanol, zinc, a-lipoic acid, genistein, coenzyme Q10, vitamin D, catechin, L-citrulline, curcumin and quercetin showed, while no effect on CVD or T2DM risk was found after supplementation with vitamin E, vitamin C, vitamin D, and selenium [152].

A 12-month single blinded multicenter randomized control trial compared 456 T2DM patients divided into a control arm taking metformin + Placebo, a parallel arm I taking metformin + Ascorbic Acid 500 mg once daily and a parallel arm II taking metformin + Acetylsalicylic Acid 100 mg. It was found that parallel arm I was twice as likely to reduce HbA1c compared to the control arm in one year and it was ten times more likely to reduce risk factors causing long-term diabetes complications compared to participants in arm II in one year; conversely, parallel arm II were seven times more effective in reducing the risk of expected CVD development over 10 years compared to arm I. Furthermore, the study showed a significant reduction in the Framingham score compared with baseline: moderate per the arm I (*p* < 0.001), low for the arm II (*p* < 0.001), however control arm showed an increased mean score sustaining ‘high risk’ [150].

An update to a systematic review and meta-analyses of systematic reviews and single randomized controlled trials (RCTs) showed no effect in preventing CVD, myocardial infarction (MI) or stroke, nor for all-cause mortality was seen for calcium, vitamin D and vitamin C [153].

A cross-sectional observational study measured vitamin C levels, assessed by high-performance liquid chromatography (HPLC), and consumption of fresh fruit and vegetables, assessed by a food frequency questionnaire, in 200 outpatients with T2DM and it showed a vitamin C deficiency in 12.2% of the subjects and that subjects with CVD had lower vitamin C levels and consumption of fresh fruit and vegetables, while there was an association between consumption of fresh fruit and vegetables and higher levels of vitamin C. It was also found that in patients with vitamin C deficiency, there were lower total and HDL-cholesterol and higher triglycerides, while vitamin C levels were significantly and progressively lower with the aggravation of carotid artery stenosis and they were significantly lower in ischemic heart disease, while a trend towards lower levels of vitamin C was seen in peripheral artery disease. With the aim of evaluating the relationship between vitamin C levels and atherosclerotic burden, the authors created a categorical variable in which 0 represented absence and 1 represented the presence of ischemic heart diseases or peripheral artery disease or cerebrovascular disease or previous carotid thromboarterectomy; they found that group with atherosclerotic conditions had significantly lower vitamin C levels. In their multivariable analysis, vitamin C was also independently and inversely associated with CVD, so consumption of vitamin C from fresh fruit and vegetables could have a protective role [151].

## 7. Potential and Limitations of Randomized Clinical Trials, Meta-Analysis and Systematic Reviews

Clinical trials and systematic reviews on Vitamin C highlight its strong mechanistic potential but reveal significant clinical limitations regarding its use in T2DM and Metabolic Syndrome. In this regard, the strengths of this study include the summarization and integration of the highest quality of evidence available on the topic (Table 1). However, limitations must be recognized. Due to the nature of the search string, some very recent randomized clinical trials or observational studies may not have been discussed. However, we checked, and the few studies found do not significantly add to the literature reviewed. Mechanistically, vitamin C’s effective antioxidant capacity (scavenging ROS and nitrogen species) and its demonstrated ability in experimental studies to improve skeletal muscle insulin sensitivity suggest a potent therapeutic role against oxidative stress, a core element of T2DM pathophysiology. However, the evidence from pooled analyses remains highly contrasting. While studies like Mason et al. [35] demonstrated a potential benefit with significant reductions in glycemic indices, particularly in longer trials (>12 weeks), other analyses, such as Khodaeian et al. [67], found no significant improvement in insulin resistance especially when vitamin C was used alone. The main limitations of the available evidence include profound methodological heterogeneity (dosing from 72 to 6000 mg/day and intervention duration from 2 weeks to 9 years), low or very low quality of meta-analyses (as assessed by AMSTAR-2 and GRADE), and the complexity introduced by vitamin C’s impaired bioavailability in chronic hyperglycemic states. Furthermore, the observation that high doses of vitamin C can paradoxically increase FBG values and the possibility of a dual antioxidant/pro-oxidant behavior further confound outcomes. Therefore, while vitamin C may improve different glycemic variables in some patient subgroups, its influence on insulin resistance remains uncertain, necessitating future adequately powered RCTs with standardized dosing regimens and extended duration to confirm long-term efficacy.

## 8. Conclusions

Vitamin C is an essential micronutrient that acts as a vital antioxidant and enzymatic cofactor in humans, although its therapeutic efficacy for chronic diseases remains complex. While Vitamin C deficiency and low circulating levels are frequently associated with increased oxidative stress, in diabetes with its comorbidities, gestational diabetes mellitus, and metabolic syndrome with higher cardiovascular risk, findings from large randomized controlled trials (RCTs) often showed low or no significant benefit from Vitamin C supplementation for preventing cardiovascular risk, improving insulin resistance (HOMA-IR), decrease HbA1C in T2DM and metabolic syndrome. This contrasting data are due to methodological heterogeneity across studies, low evidence quality, and different dosing utilized. Therefore, at this time, combining micronutrient supplementation and a holistic dietary approach with effective weight loss interventions may be synergistic in improving glucose tolerance, insulin resistance, and dyslipidemia and remains the most effective strategy for mitigating metabolic and cardiovascular risks in type 2 diabetes and metabolic syndrome. Furter studies using larger sample sizes and longer supplementation periods that are powered to stratify effects on the basis of the baseline glycemic control required necessary to confirm the beneficial effects of vitamin C supplementation on diabetes and metabolic syndrome management.

## Figures and Tables

**Table 1:** Synthesis of meta-analyses and systematic reviews on the effectiveness of various dosages of vitamin C administration in pathologic conditions analyzed.

Author	Design	Duration	Participants	Dose of Vitamin C	Results
**VITAMIN C AND INSULIN RESISTANCE (IR)**					
Yi Chai et al. [70]	UR (14 SR with 162 primary RCTs)	From 14 days to 9 years	Target population were patients with T2DM; also, individuals with other metabolic disorders including obesity, polycystic ovary syndrome, metabolic Syndrome Number not available	From 72 to 6000 mg/day	Vitamin C supplementation could reduce FBG, with pooled effect sizes ranging from −20.59 (95% CI: −40.77 to −0.4) to −0.44 (95% CI: −0.81 to −0.07), and statistically more significant positive effect with durations longer than 30 days ranging from −0.53 (95% CI: −0.97 to −0.10); reduction in HbA1c with pooled effect sizes ranging from −0.54 (95% CI: −0.9, −0.17) to −0.37 (95% CI: −0.57, −0.17); no effect on insulin.
Khodaeian et al. [68]	SR (14 RCTs)	From 4 to 16 weeks	735 patients with T2DM without severe diabetes complications	From 800 to 1000 mg	Vitamin C-only or in combination with VE supplements did not cause significant improvement in HOMA index (SMD: −0.150, 95% CI: 0 494 to 0.194),
Fong et al.[69]	UR (13 studies)	From 2 weeks to 1 year	6409 patients of any health status and 1574 patients withT2DM for vitamin C group	From 500 to 3000 mg	Vitamin C supplementation caused significant reductions in FBG (MD −0.74 mmol/L), HbA1c (MD −0.54%), and postprandial glucose (MD −0.95 mmol/L). These effects were more evident in trials of >12 weeks duration and in individuals with elevated baseline HbA1c, suggesting greater benefit in poorly controlled patients.
**VITAMIN C AND TYPE 2 DIABETES MELLITUS (T2DM)**					
AW Ashor et al. [33]	SR and MT (22 RCTs)	Median duration: 30 days	937 participants (13 trials with T2DM)	From 500 to 2000 mg	Subgroup analyses revealed that in T2DM, vitamin C intake led to a reduction in fasting plasma glucose in interventions > 30 days. No significant changes in postprandial insulin concentration. No significant effect on HbA1c.
S. Nosratabadi et al. [88]	SR and MT (22 RCTs)	2 weeks	1447 participants with T2DM	From 200 to 2000 mg	Vitamin C supplementation was associated with a reduction in fasting blood glucose, fasting insulin levels, and HbA1c compared to untreated control groups. Decrease in HbA1c was pronounced in an intervention duration of ≥12 weeks and daily doses of ≥1000 mg.
A-M. Lampousi et al. [84]	SR and MT (25 POSs and 15 RCTs)	There is not a single fixed duration—the follow-up periods varied depending on each included study	27 studies investigated T2DM. The exact total number of T2D cases is distributed across these individual studies, but it is not a single number for the whole meta-analysis	The meta-analysis found that the lowest risk of T2D was observed at an intake of ~70 mg/day of vitamin C from diet.	Nonlinear dose–response relationship between dietary vitamin C and T2DM; an intake of 70 mg/day was associated with a 24% lower risk of type 2 diabetes (RR: 0.76; 95% CI: 0.61, 0.95)
**VITAMIN C AND GESTATIONAL DIABETES (GDM)**					
Zhou et al. [91]	SR and MT (13 Case–Control studies and 2 Cohort studies)	From pre-pregnancy to post-pregnancy	10,131 subjects, of which 1304 were diagnosed with GDM	Dietary intake of Vitamin C (not including Vitamin C supplements) or blood Vitamin C concentration.	Risk of GDM is higher in women with lower vitamin C exposure, even if there was statistically significant heterogeneity among the included studies.
**VITAMIN C AND DIABETIC COMPLICATIONS**					
Kedzierska-Kapuza et al. [97]	SR (9 RCTs, 2 meta-analyze, 3 prospective studies, 2 retrospective studies, 2 cross-sectional)	There was no single study duration, since the analysis included 18 different studies published between 2012–2022	32.000 participants across the included primary studies	Low-to-moderate doses (250–500 mg), given either after dialysis or daily orally	The inclusion of vitamin C infusions can be used as supportive therapy
Kurian et al. [154] [Shilia Jacob Kurian, Tejaswini Baral, Mazhuvancherry K. Unnikrishnan, Ruby Benson, Murali Munisamy, Kavitha Saravu, Gabriel Sunil Rodrigues, Mahadev Rao, Amit Kumar7 and Sonal Sekhar Miraj]	SR (9 RCTs, 12 cross-sectional studies, 7 cohort studies, 9 case–control studies)	The database search was conducted initially in July 2021 and updated on 21 October 2021	1.433 participants with diabetic foot ulcers	From 250 to 500 mg daily	Vitamin C deficiency is common in DFU and linked to poor outcomes; nutritional assessment and supplementation should be integrated into DFU care
Shah et al. [155]	SR (3 interventional studies, 17 prospective studies, 29 cross-sectional studies, 5 case–control studies)	There was no single study duration, since the analysis included 54 different studies published between January 1967 to May 2022	Across the 54 included studies, the total number of participants is approximately 189.066	Dietary intake around 180 mg of vitamin C	A correct daily intake of vitamin C might be associated with a protective effect
**VITAMIN C AND METABOLIC SYNDROME (MetS)**					
Guo et al. [39]	MT (28 OS)	There was no single study duration, since the analysis included 28 different studies published between 2003–2021	From 143 to 27,656 for a total number of 110,771	Dietary intake and circulating concentrations of vitamin C in relation to MetS prevalence	They found that, among individuals with MetS, were present both a lower dietary of vitamin C levels compared with controls (SMD = –0.04; 95% CI: –0.08 to –0.01) and reduced circulating levels of vitamin C relative to controls (SMD = −0.82; 95% CI: −1.24 to −0.40), both of which negatively associated with MetS.
**VITAMIN C AND ARTERIAL HYPERTENSION**					
De Paula et al. [130]	SR and MT (11 RCTs)	From 3 to 52 weeks	723 patients with T2DM	From 500 mg to 1500 mg/day	Vitamin C has not effect on SBP (WMD − 3.93 mmHg; p = 0.478). However, there was a reduction of −2.88 mmHg (p = 0.020) in DBP compared with the control groups
Guan et al. [129]	SR and MT (8 RCTs)	From 4 to 24 weeks	614 patients with essential hypertension	From 300 to 1000 mg/day	There was a significant difference in the reduction in SBP (WMD = 4.09; *p* < 0.001) and of DBP (WMD = 2.30; *p* = 0.02) between the groups. Furthermore, significant difference in the SBP (WMD = −3.75, *p* = 0.003) and DBP (WMD= 3.29, *p* = 0.02) was seen also for the subgroup with an age ≥60 years and that with ≥35 participants. In the subgroup analysis, with regard to study duration ≥6 weeks, result for SBP was statistically significant different (WMD = 4.77; *p* < 0.001). Furthermore, with the use of a dose of Vitamin C ≥500mg/day, there was a statistically significant reduction on SBP (WMD = 5.01; *p* = 0.005).
Mason et al. [36]	SR and MT (28 RCTs)	From 2 weeks to 1 year	1574 patients with T2DM	From 200 to 3000 mg/day	Vitamin C determined a significant and statistically reduction on SBP (mean difference 26.27 mmHg) with moderate evidence certainty, and on DBP (23.77 mmHg) with very low evidence certainty.
**VITAMIN C AND LIPID PROFILE**					
Namkhah et al. [132]	SR and MT (11 parallel studies and 4 cross-over studies)	From 2 to 48 weeks	872 patients with T2DM	From 200 and 2000 mg/dL	Vitamin C decreases as TG and TC, but failed to significantly change LDL and HDL
Gillani et al. [150]	Single blind multicenter RCT	12 month	456 patients with DMT2	Recruited patients were randomly assigned to three groups: - Control arm (metformin + Placebo) which received usual metformin (Glucophage) with Placebo once daily (blinded). - Parallel arm I (metformin + Ascorbic Acid (ACA) 500 mg) which received Ascorbic Acid 500 mg once daily in addition to usual metformin (Glucophage) dose - Parallel arm II (metformin + Acetylsalicylic Acid (ASA) 100 mg) which received a dose of Acetylsalicylic Acid 100 mg once daily in addition to usual metformin (Glucophage) dose	Significant reduction in LDL-c, TG and CT with ascorbic acid group compared to control group
Dludla et al. [137]	SR (21 RCTs)	4–6 weeks	7688 patients with T2DM, T1DM or MetS	1000 mg/day or 500 mg twice daily	Vitamin C intake reduces cholesterol levels and improving metabolic function in patients with diabetes or MetS.
**VITAMIN C AND OBESITY**					
Mazaheri-Tehrani et al. [144]	SR (47) and MT (37) (Cross-Sectional studies, Case–Control Studies and Cohort Studies)	Data not available	49,401 healthy and sick people	Dietary or Circulating vitamin C levels	Most studies reported an inverse correlation between BMI/WC and serum vitamin C levels
Gillani et al. [150]	Single blind multicenter RCT	12 month	456 patients with DMT2	Recruited patients were randomly assigned to three groups: - Control arm (metformin + Placebo) which received usual metformin (Glucophage) with Placebo once daily (blinded). - Parallel arm I (metformin + Ascorbic Acid (ACA) 500 mg) which received Ascorbic Acid 500 mg once daily in addition to usual metformin (Glucophage) dose - Parallel arm II (metformin + Acetylsalicylic Acid (ASA) 100 mg) which received a dose of Acetylsalicylic Acid 100 mg once daily in addition to usual metformin (Glucophage) dose	Any significant change (reduction or increase) to anthropometric values both within the group and intergroup (compare to control)
**VITAMIN C AND CARDIOVASCULAR DISEASES (CVD)**					
An et al. [152]	SR and MT (884 RCTs)	From 3 to 9.4 years	883.627 participants	500 mg/day	Vitamin C showed no effect on CVD
Gillani et al. [150]	Single blind multicenter RCT	12 month	456 patients with T2DM	Recruited patients were randomly assigned to three groups: - Control arm (metformin + Placebo) which received usual metformin (Glucophage) with Placebo once daily (blinded). - Parallel arm I (metformin + Ascorbic Acid (ACA) 500 mg) which received Ascorbic Acid 500 mg once daily in addition to usual metformin (Glucophage) dose - Parallel arm II (metformin + Acetylsalicylic Acid 100 mg) which received a dose of Acetylsalicylic Acid 100 mg once daily in addition to usual metformin (Glucophage) dose.	Parallel arm I was twice more likely effective in the reduction on HbA1c than control arm (*p* < 0.001). Parallel arm I was ten times more likely effective on the reduction on risk factors of long-term diabetes complications than participants of arm II (*p* < 0.001). Parallel arm II patients were seven times more effective on the reduction on the risk of expected CVD development in 10 years than arm I (*p* < 0.001).
Jenkins et al. [153]	Update of the previous 2018 SR and MT (35 newly included RCTs)	Data not available	Data not available	Data not available	No effect on the prevention of MI, CVD or stroke, nor for all-cause mortality was seen for Vitamin C.

WMD: Weighted Mean Difference; MetS: Metabolic Syndrome; SR: Systematic Review; MT: Meta-analysis; RCT: Randomized Controlled Study; T2DM: Type 2 Diabetes Mellitus; T1DM: Type 1 Diabetes Mellitus; GDM: Gestational Diabetes Mellitus; SBP: Systolic Blood Pressure; DBP: Diastolic Blood Pressure; CVD: Cardiovascular Diseases; MI: Myocardial Infarction; UR: umbrella review; POS: prospective observational study; FBG: fasting blood glucose; HbA1c: glycated hemoglobin; HOMA: Homeostasis Model Assessment-insulin resistance; VE: vitamin E; LDL: Low-Density Lipoprotein; HDL: High-Density Lipoprotein; TG: triglycerides; CT: total cholesterol; BMI: Body Mass Index; WC: Waist Circumferences; DFU: Diabetic foot ulcers; OS: Observational Studies.

## Data Availability

No new data were created or analyzed in this study. Data sharing is not applicable to this article.
